# Admission blood gas, platelet, and inflammatory parameters associated with intraventricular hemorrhage in preterm infants: a retrospective case-control study

**DOI:** 10.1515/med-2026-1521

**Published:** 2026-09-07

**Authors:** Zeynep Alara Saltık, Hakan Aylanc

**Affiliations:** Faculty of Medicine, Child Health and Diseases, Canakkale Onsekiz Mart University, Canakkale, Türkiye; Faculty of Medicine, Child Health and Diseases, Department of Neonatology, Canakkale Onsekiz Mart University, Canakkale, Türkiye

**Keywords:** intraventricular hemorrhage, lactate, mean platelet volume, neutrophil-to-lymphocyte ratio, preterm infants

## Abstract

**Objectives:**

This study aimed to evaluate the association between admission blood gas, platelet, and inflammatory parameters and the presence of IVH.

**Methods:**

This retrospective case-control study included 356 preterm infants admitted to a tertiary neonatal intensive care unit between 2016 and 2025, including 165 infants with IVH and 191 without IVH. Initial blood gas and complete blood count parameters were analyzed. Multivariable logistic regression was used to identify independent factors associated with IVH.

**Results:**

Lower gestational age was significantly associated with IVH presence and severity (p<0.001). Lactate levels were higher in the IVH group (3.10 vs. 2.60 mmol/L; p=0.013). In the exploratory integrated model including gestational age, lactate, platelet count, MPV, and the N/L ratio, lactate showed a borderline association with IVH (OR=1.099, 95 % CI: 0.995–1.214; p=0.063), whereas lower platelet count, lower MPV, and higher N/L ratio remained independently associated with IVH.

**Conclusions:**

Lower platelet counts, lower MPV values, and higher N/L ratios were independently associated with IVH in the exploratory integrated model, while admission lactate showed a borderline association after adjustment for gestational age and hematological parameters. These findings should be interpreted as early associations rather than validated predictive biomarkers, and prospective studies with standardized cranial ultrasonography and external validation are needed.

## Introduction

Prematurity remains one of the leading causes of neonatal morbidity and mortality today. According to World Health Organization data, approximately 15 million babies are born prematurely each year, accounting for about 10 % of all live births [1]. It has been reported that preterm birth rates in Turkey remain high, similar to the global average, and that this situation continues to be a significant public health issue [2]. Due to immature organ systems, many systemic complications can develop in preterm infants. In particular, the central nervous system is extremely sensitive to hemodynamic fluctuations and hypoxic-ischemic events, which is why neurological morbidity presents as a major problem in the preterm population [3].

Germinal matrix hemorrhage (GMH) and intraventricular hemorrhage (IVH) are the most common types of brain injury in preterm infants and are observed with higher frequency, particularly in infants with extremely low gestational age and low birth weight. The dense vascularization of the germinal matrix, the structural fragility of blood vessel walls, and inadequate cerebral autoregulation increase susceptibility to hemorrhage in response to sudden changes in cerebral blood flow [3]. Despite advances in neonatal intensive care practices, GMH-IVH remains a significant perinatal complication associated with high mortality rates and long-term neurological sequelae.

Blood gas parameters assessed in the first hours of life in preterm infants provide important information regarding tissue oxygenation and perfusion status; meanwhile, hematological parameters may serve as indicators of inflammatory response, hematological status, and coagulation balance. Although markers such as lactate, glucose, hemoglobin, platelet count, and platelet indices have been reported to be associated with IVH, the combined relationship of admission blood gas and hematological parameters with IVH has not been fully clarified. Therefore, this study aimed to evaluate the association between initial blood gas and hematological parameters obtained at admission and the presence of IVH in preterm infants monitored in the neonatal intensive care unit.

## Methods

This retrospective case-control study reviewed the medical records of 3,293 neonates admitted to the NICU of Canakkale Onsekiz Mart University (COMU) Hospital between 2016 and 2025. Patient records were screened through the hospital electronic database. Among 1,433 infants diagnosed with prematurity (ICD-10 code P07.3), 177 infants were identified as having non-traumatic intracranial hemorrhage (ICD-10 codes P52.0–P52.3). Medical records of these infants were reviewed individually. After applying the inclusion and exclusion criteria, 165 infants with IVH were eligible for analysis and constituted the case group. The control group consisted of 191 preterm infants without IVH who met the same eligibility criteria, had available admission blood gas and complete blood count data, and were admitted during the same study period. Controls were selected from the NICU database according to data availability after applying the same exclusion criteria used for the IVH group.

The inclusion criteria for the overall study population were: gestational age <37 weeks, availability of admission blood gas and complete blood count results, and sufficient clinical data. For the case group, the presence of IVH was required, whereas controls were preterm infants without IVH who met the same eligibility criteria. Infants born outside the study center, transferred from another institution without admission laboratory data, transferred for advanced diagnostic or therapeutic procedures, or who died within the first 24 h of life were excluded from the study. Consequently, 12 of the 177 initially identified IVH patients were excluded, leaving 165 patients for the final analysis.

Data were obtained from digital patient records of the NICU and the Department of Obstetrics and Gynecology. The following variables were collected: gestational age, birth weight, presence and grade of IVH according to the Papile classification, hematological parameters [white blood cell count (WBC), hemoglobin (Hgb), platelet count (PLT), mean platelet volume (MPV), neutrophil count (Neu), lymphocyte count (Lym), eosinophil count (EOS), and neutrophil-to-lymphocyte ratio (N/L)], and admission blood gas parameters [pH, partial pressure of carbon dioxide (pCO_2_), hemoglobin (Hgb), hematocrit (Hct), carboxyhemoglobin (COHb), glucose, lactate, and standard base excess (SBE)]. Admission blood gas and complete blood count results were obtained from routine laboratory records at NICU admission. Blood gas analyses were performed using the hospital’s routine blood gas analyzers; the currently used analyzer is the Radiometer ABL90 FLEX, although analyzer platforms may have changed during the 2016–2025 study period. Complete blood counts were analyzed from EDTA-anticoagulated blood samples using the hospital’s routine hematology analyzers. Laboratory analyses were performed according to the hospital laboratory’s routine procedures and internal quality control practices. Because of the retrospective design, blood gas sampling source was not standardized and could include umbilical or peripheral venous/arterial samples according to clinical circumstances. Exact handling time and time from sampling to analysis could not be verified for all patients. Whether all samples were obtained before therapeutic interventions could not be verified retrospectively.

Cranial ultrasonography examinations were requested as transfontanel ultrasonography and performed as part of routine neonatal care by radiology residents. IVH grade was obtained from routine radiology reports and classified according to the Papile classification. The ultrasound assessors were not specifically involved in the study and were not aware of the study hypothesis; however, formal blinding to laboratory findings could not be verified retrospectively. Interobserver agreement was not evaluated because examinations were interpreted as part of routine clinical practice by a single radiology resident.

The study was approved by the Çanakkale Onsekiz Mart University Non-Interventional Clinical Research Ethics Committee (Approval No. 2023/249, December 27, 2023) and was conducted in accordance with the principles of the Declaration of Helsinki (revised in 2013). The requirement for informed consent was waived by the Institutional Review Board due to the retrospective nature of the study.

Statistical analyses were performed using JAMOVI version 2.7.15 and IBM SPSS Statistics 26. Descriptive statistics presented categorical variables as counts and percentages [n (%)], while continuous variables were presented as mean ± standard deviation or median (minimum–maximum) values, depending on their distribution characteristics. The normality of continuous variables was assessed using the Kolmogorov–Smirnov and Shapiro–Wilk tests. For comparisons between two groups of continuous variables that did not follow a normal distribution, the Mann–Whitney U test was used; for comparisons involving three or more groups, the Kruskal–Wallis test was used. For binary comparisons of continuous variables determined to follow a normal distribution, the independent samples *t*-test was applied. For comparisons between categorical variables, the Pearson chi-square test was used; in cases where the expected cell count was less than 5, Fisher’s exact test was preferred. Multivariable logistic regression analysis was performed to identify factors associated with the presence of IVH. Separate multivariable logistic regression models were constructed for blood gas parameters and hematological parameters. Gestational age and birth weight were included as clinically relevant covariates where appropriate. Results were reported as odds ratios (OR) and 95 % confidence intervals (CI). In all statistical analyses, a p-value of <0.05 was considered statistically significant. Additional analyses were performed to address model integration and collinearity. Gestational age and birth weight were evaluated for collinearity using Pearson correlation analysis. An exploratory integrated multivariable logistic regression model was constructed including gestational age, lactate, platelet count, MPV, and the N/L ratio. Because gestational age and birth weight were strongly correlated, both variables were not entered simultaneously into the integrated model. Gestational age was retained as the principal marker of prematurity. Model calibration was evaluated using the Hosmer–Lemeshow test, and discrimination was assessed using receiver operating characteristic analysis.

## Results

**Figure 1:**
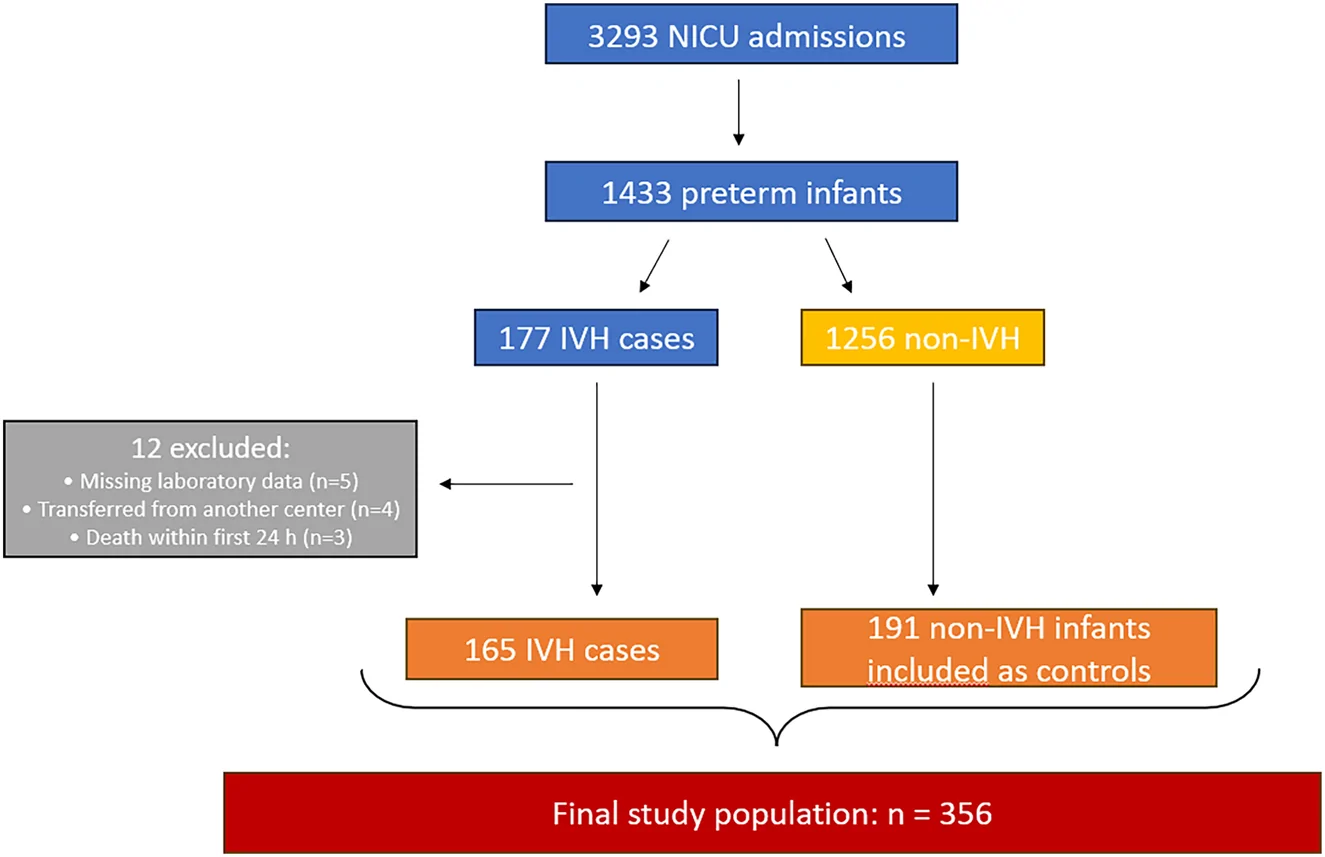
Flow diagram of patient selection and study population.

Between 2016 and 2025, a total of 3,293 neonates were admitted to the NICU of COMU Hospital, of whom 1,433 (43.5 %) were preterm infants. Among these infants, 177 were initially identified as having non-traumatic intracranial hemorrhage (ICD-10 codes P52.0–P52.3). After applying the inclusion and exclusion criteria, 165 infants with IVH and 191 preterm infants without IVH were included in the final analysis. The flow diagram of patient selection and study population is presented in Figure 1.

The gestational ages of the 356 infants included in the study ranged from 23 to 36 weeks. When gestational age was evaluated by individual gestational weeks, a statistically significant association was observed between gestational age and the presence of IVH (χ^2^=39.564, df=14, p<0.001). The mean gestational age was significantly lower in infants with IVH than in those without IVH (30.42 ± 3.34 vs. 32.16 ± 2.78 weeks, p<0.001). IVH was detected in 60.7 % (n=99) of the 163 infants born before 32 weeks, compared with 34.2 % (n=66) of the 193 infants born at or after 32 weeks (χ^2^=25.030, p<0.001). Infants born before 32 weeks had approximately threefold higher odds of IVH than those born at or after 32 weeks (OR=2.98; 95 % CI: 1.93–4.59). In logistic regression analysis, gestational age remained significantly associated with IVH (OR=0.780; 95 % CI: 0.689–0.884; p<0.001), with each additional week of gestation associated with an approximately 22 % reduction in the odds of IVH.

When birth weights were evaluated, the mean birth weight was significantly lower in infants with IVH than in those without IVH (1,593.85 ± 661.76 g vs. 1,827.98 ± 574.91 g, p<0.001). IVH was detected in 59.3 % (n=80) of infants with a birth weight <1,500 g, compared with 38.5 % (n=85) of those with a birth weight ≥1,500 g (χ^2^=14.58, p<0.001). Risk analysis demonstrated that infants with a birth weight <1,500 g had a 1.54-fold higher risk of IVH than those with a birth weight ≥1,500 g (RR=1.541; 95 % CI: 1.239–1.915).

Both 1-min and 5-min Apgar scores were significantly lower in infants with IVH. The mean 1-min Apgar score was 5.90 ± 2.02 in the IVH group and 6.98 ± 1.56 in the non-IVH group (p<0.001). Similarly, the mean 5-min Apgar score was 7.31 ± 1.56 in the IVH group and 8.23 ± 1.31 in the non-IVH group (p<0.001).

The overall cohort included 193 male infants (54.2 %) and 163 female infants (45.8 %). IVH was observed in 84 male infants (43.5 %) and 81 female infants (49.7 %). There was no statistically significant difference in IVH occurrence according to sex (p=0.245).

Multiple pregnancy was present in 131 infants, including 125 twins and 6 triplets. IVH was observed in 110 of 225 infants from singleton pregnancies (48.9 %) and in 55 of 131 infants from multiple pregnancies (42.0 %). There was no statistically significant association between multiple pregnancy and IVH occurrence (p=0.208).

Several antenatal, perinatal, and neonatal variables were compared according to IVH status and are presented in Table 2. Infants with IVH had significantly lower gestational age, lower birth weight, and lower 1-min and 5-min Apgar scores. Vaginal delivery, surfactant administration, maternal chorioamnionitis, need for resuscitation at birth, hemodynamically significant PDA, hypotension requiring inotropic support, hypoglycemia, proven sepsis, and NEC were significantly associated with IVH in univariate analyses. In contrast, sex, multiple pregnancy, and antenatal steroid exposure were not significantly associated with IVH occurrence in univariate analyses.

The grade of IVH was determined based on the initial cranial ultrasound. Among the 165 infants with IVH, 76.4 % (n=126) had grade I IVH, 15.2 % (n=25) had grade II, 4.8 % (n=8) had grade III, and 3.6 % (n=6) had grade IV. The mean postnatal age at the first cranial ultrasound examination was 3.73 ± 3.3 days.

When IVH severity was evaluated according to prematurity subgroups, severe IVH (grade III–IV) was observed more frequently in infants with lower gestational age. Among infants with IVH, severe IVH was observed in 21.9 % of extremely preterm infants, 6.0 % of very preterm infants, 9.7 % of moderate preterm infants, and 0 % of late preterm infants. This difference was statistically significant (χ^2^=11.236, p=0.011).

As shown in Table 1, IVH was detected in 72.7 % of extremely preterm infants, 56.3 % of very preterm infants, 34.8 % of moderate preterm infants, and 33.7 % of late preterm infants. The frequency of IVH increased significantly with decreasing gestational age (χ^2^=28.541, p<0.001) (Table 2).

**Table 1:** Presence of IVH according to prematurity groups.

	No IVH n, %	IVH n, %	Total number, %
23–27 weeks	12 (27.3)	32 (72.7)	44 (100)
28–31 weeks	52 (43.7)	67 (56.3)	119 (100)
32–34 weeks	58 (65.2)	31 (34.8)	89 (100)
35–36 weeks	69 (66.3)	35 (33.7)	104 (100)
**Total number**	**191 (53.7)**	**165 (46.3)**	**356 (100)**

**Table 2:** Clinical characteristics of preterm infants according to IVH status.

		IVH status		p-Value
		Non-IVH	IVH group	
		Mean ± SD	Mean ± SD	
Gestational age, weeks		32.16 ± 2.78	30.42 ± 3.34	<0.001
Birth weight, grams	1,827.98 ± 574.91		1,593.85 ± 661.76	<0.001
1-min apgar score		6.98 ± 1.56	5.9 ± 2.02	<0.001
5-min apgar score	8.23 ± 1.31		7.31 ± 1.56	<0.001

		**n, %**	**n, %**	**p-Value**

Antenatal steroid	None	87 (49.2)	90 (50.8)	0.071
	Given once	20 (47.6)	22 (52.4)	
	Given twice	84 (61.3)	53 (38.7)	

Surfactant administration	No	137 (61.7)	85 (38.3)	<0.001
	Yes	54 (40.3)	80 (59.7)	

Maternal chorioamnionitis	No	184 (57.0)	139 (43.0)	<0.001
	Yes	7 (21.2)	26 (78.8)	

Sex	Male	109 (56.5)	84 (43.5)	0.245
	Female	82 (50.3)	81 (49.7)	
Pregnancy type	Singleton	115 (51.1)	110 (48.9)	0.208
	Multiple	76 (58.0)	55 (42.0)	

Delivery mode	Vaginal	10 (21.2)	37 (78.8)	<0.001
	C/S	181 (58.5)	128 (41.5)	

Need for resuscitation at birth	No	173 (59.2)	119 (40.8)	<0.001
	Yes	18 (28.1)	46 (71.9)	

Hemodynamically significant PDA	No	165 (58.7)	116 (41.3)	<0.001
	Yes	26 (34.7)	49 (65.3)	

Hypotension requiring inotropic support	No	162 (62.7)	96 (37.3)	<0.001
	Yes	29 (29.5)	69 (70.5)	

Hypoglycemia	No	149 (57.1)	112 (42.9)	0.031
	Yes	42 (44.2)	53 (55.8)	

Proven sepsis	No	167 (58.0)	121 (42.0)	0.001
	Yes	24 (35.3)	44 (64.7)	

Necrotizing enterocolitis	No	155 (57.4)	115 (42.6)	0.012
	Yes	36 (41.9)	50 (58.1)	

Data are presented as mean ± SD, or n (%). Percentages represent row percentages. IVH, intraventricular hemorrhage; PDA, patent ductus arteriosus; NEC, necrotizing enterocolitis; C/S, cesarean section.

When initial blood gas parameters were evaluated, no statistically significant differences were observed between infants with and without IVH in terms of pH, pCO_2_, hemoglobin, hematocrit, or SBE values (all p>0.05). Glucose and lactate levels were significantly higher in infants with IVH (p=0.022 and p=0.013, respectively). COHb values differed statistically between groups (p=0.045); however, because implausible negative and extremely high values were present, COHb results were interpreted descriptively and were not included in multivariable models. Detailed comparisons of blood gas parameters are presented in Table 3. The median glucose level was 68 (6–196) mg/dL in the IVH group and 59 (12–181) mg/dL in the non-IVH group. Similarly, median lactate levels were higher in infants with IVH than in those without IVH [3.10 (0.80–22.00) vs. 2.60 (0.70–10.80) mmol/L, respectively].

**Table 3:** Comparison of initial blood gas and complete blood count parameters according to the presence of IVH.

Parameter	No IVH (median, min–max)	IVH (median, min–max)	p-Value
pH	7.27 (6.99–7.47)	7.26 (6.83–7.54)	0.641
pCO_2_, mmHg	52.7 (23.7–87.4)	50.9 (16.2–95.4)	0.121
Blood gas Hgb, g/dL	17.5 (11.9–25.9)	17.0 (5.9–25.6)	0.102
Hct, %	53.5 (36.7–78.8)	51.9 (18.7–77.9)	0.108
COHb, %	1.40 (0.10–85.50)	1.30 (−0.30 to 101.00)	0.045
Glucose, mg/dL	59 (12–181)	68 (6–196)	0.022
Lactate, mmol/L	2.60 (0.70–10.80)	3.10 (0.80–22.00)	0.013
SBE	−3.20 (−14.8 to 5.2)	−3.30 (−46.0 to 4.8)	0.133
WBC (×10^3^/µL)	10.6 (3.87–72.70)	10.7 (2.99–106.50)	0.843
CBC Hgb, g/dL	16.3 (11.6–22.1)	15.5 (4.6–24.9)	0.001
PLT (×10^3^/µL)	261 (61–441)	238 (78–493)	0.008
MPV, fL	10.0 (6.5–12.6)	8.5 (6.4–18.5)	<0.001
Neu (×10^3^/µL)	3.88 (0.63–44.30)	4.83 (0.61–92.20)	0.092
Lym (×10^3^/µL)	4.89 (1.36–27.00)	4.75 (1.00–21.00)	0.177
N/L ratio	0.84 (0.10–7.25)	1.07 (0.07–9.71)	0.013
EOS (×10^3^/µL)	0.24 (0.00–1.91)	0.20 (0.00–1.30)	0.034

p-Value, Mann–Whitney U test.

A multivariable blood gas model adjusted for gestational age and birth weight was performed to evaluate whether admission blood gas parameters were associated with the presence of IVH. The model was statistically significant (p<0.001), and model fit was adequate according to the Hosmer–Lemeshow test (p=0.613).

In the blood gas model, lactate level was independently associated with the presence of IVH (OR=1.12; 95 % CI: 1.02–1.23; p=0.018), indicating that each 1 mmol/L increase in lactate was associated with an approximately 12 % increase in the odds of IVH.

Although glucose levels were associated with IVH in univariate analysis, they were not identified as an independent factor in the multivariable model.

When hematological parameters were compared between infants with and without IVH, statistically significant differences were observed in several markers. Significant differences were found in hemoglobin, platelet count, MPV, eosinophil count (EOS), and the neutrophil-to-lymphocyte (N/L) ratio (all p<0.05). In the IVH group, median Hgb, PLT, and MPV values were significantly lower, whereas the N/L ratio was significantly higher. The median eosinophil count was also lower in infants with IVH. No significant differences were observed in total white blood cell, neutrophil, or lymphocyte counts (all p>0.05). Detailed hematological findings are presented in Table 3.

Hemoglobin levels were significantly lower in infants with IVH than in those without IVH [15.5 (4.6–24.9) vs. 16.3 (11.6–22.1) g/dL, p=0.001]. Hemoglobin levels also differed significantly across IVH grades (H=20.456, p<0.001), with lower values observed in higher-grade IVH.

Platelet counts were significantly lower in infants with IVH [238 (78–493) vs. 261 (61–441) × 10^3^/µL, p=0.008], although no significant differences were observed across IVH grades (H=8.954, p=0.062).

MPV values were significantly lower in infants with IVH [8.5 (6.4–18.5) vs. 10.0 (6.5–12.6) fL, p<0.001] and showed a significant decrease with increasing IVH grade (H=60.510, p<0.001).

The N/L ratio was significantly higher in infants with IVH [1.07 (0.07–9.71) vs. 0.84 (0.10–7.25), p=0.013], whereas eosinophil counts were significantly lower [0.20 (0–1.30) vs. 0.24 (0–1.91), p=0.034].

In the multivariable logistic regression analysis performed to identify independent hematological factors associated with IVH, the model was statistically significant (Omnibus test: χ^2^=94.825; p<0.001). The explanatory power of the model was Nagelkerke R^2^=0.314, and model fit was adequate according to the Hosmer–Lemeshow test (p=0.120).

Platelet count (OR=0.995; 95 % CI: 0.991–0.999; p=0.006), MPV (OR=0.589; 95 % CI: 0.482–0.719; p<0.001), and the N/L ratio (OR=1.265; 95 % CI: 1.012–1.580; p=0.039) were independently associated with IVH. Hemoglobin level and eosinophil count were not independently associated with IVH in the multivariable analysis. The results of the logistic regression analysis are presented in Table 4.

**Table 4:** Multivariable logistic regression analysis of factors associated with IVH.

Variable	B	S.E.	Wald	df	p-Value	OR (ExpB)	Lower 95 % CI	Upper 95 % CI
Hgb	−0.089	0.066	1.806	1	0.179	0.915	0.803	1.042
PLT	−0.005	0.002	7.631	1	0.006	0.995	0.991	0.999
MPV	−0.530	0.102	27.098	1	<0.001	0.589	0.482	0.719
EOS	0.474	0.534	0.789	1	0.374	1.607	0.564	4.574
N/L ratio	0.235	0.114	4.269	1	0.039	1.265	1.012	1.580

B, Regression coefficient; S.E., Standard error; Wald, Wald statistic; df, degrees of freedom; OR, odds ratio; CI, confidence interval.

Gestational age and birth weight were strongly correlated (r=0.817, p<0.001). Therefore, birth weight was not entered into the exploratory integrated multivariable model together with gestational age. In the integrated model including gestational age, lactate, platelet count, MPV, and the N/L ratio, gestational age (OR=0.849; 95 % CI: 0.785–0.919; p<0.001), platelet count (OR=0.996; 95 % CI: 0.992–0.999; p=0.013), MPV (OR=0.582; 95 % CI: 0.481–0.704; p<0.001), and the N/L ratio (OR=1.239; 95 % CI: 1.006–1.525; p=0.044) remained independently associated with IVH. Lactate showed a borderline association but did not reach statistical significance in this integrated model (OR=1.099; 95 % CI: 0.995–1.214; p=0.063). The model showed acceptable calibration according to the Hosmer–Lemeshow test (χ^2^=8.377, p=0.398) and acceptable discrimination, with an area under the ROC curve of 0.780. The overall correct classification rate was 72.5 % (Table 5).

**Table 5:** Exploratory integrated multivariable logistic regression model for factors associated with IVH.

Variable	OR	95 % CI	p-Value
Gestational age	0.849	0.785–0.919	<0.001
Lactate	1.099	0.995–1.214	0.063
Platelet count	0.996	0.992–0.999	0.013
MPV	0.582	0.481–0.704	<0.001
N/L ratio	1.239	1.006–1.525	0.044

OR, odds ratio; CI, confidence interval; MPV, mean platelet volume; N/L, neutrophil-to-lymphocyte ratio.

## Discussion

Advances in neonatal care have improved the survival of extremely preterm infants; however, IVH remains one of the most important complications associated with prematurity. Previous studies have consistently shown that the incidence of IVH increases as gestational age and birth weight decrease [4].

In our study, infants who developed IVH had significantly lower gestational ages than those without IVH (non-IVH: 32.16 ± 2.78 weeks; IVH: 30.42 ± 3.34 weeks). In addition, infants born before 32 weeks of gestation had approximately threefold higher odds of developing IVH compared with those born at or after 32 weeks. Multivariable analysis further demonstrated an inverse association between gestational age and IVH, with each additional week of gestation associated with an approximately 22 % reduction in the odds of IVH. These findings are consistent with previous studies demonstrating that the incidence of IVH increases as gestational age decreases. Rysavy et al. reported substantially higher rates of severe IVH among extremely preterm infants, while Ancel et al. demonstrated a progressive increase in IVH incidence with decreasing gestational age [4], 5]. The structural fragility of the germinal matrix, immature cerebral autoregulation, and increased susceptibility to hemodynamic fluctuations are considered key mechanisms underlying the increased vulnerability of extremely preterm infants to IVH.

In our study, infants who developed IVH had significantly lower birth weights than those without IVH (1,593.85 ± 661.76 g vs. 1,827.98 ± 574.91 g). In addition, infants with a birth weight below 1,500 g had 1.54-fold higher risk of developing IVH compared with those weighing 1,500 g or more. These findings are consistent with previous reports demonstrating an increased burden of IVH among very low birth weight infants. Bolisetty et al. reported an IVH prevalence exceeding 30 % in very low birth weight infants, while Payne et al. demonstrated significantly higher rates of severe IVH among infants weighing less than 1,500 g [6], 7]. Taken together, these findings suggest that low birth weight is strongly associated with IVH and that very low birth weight infants represent a particularly vulnerable group requiring close clinical surveillance.

In our study, the majority of IVH cases were classified as Grade 1 (76.4 %) and Grade 2 (15.2 %), whereas severe IVH was less common, with Grade 3 and Grade 4 disease accounting for 4.8 and 3.6 % of cases, respectively. This distribution is consistent with previous reports in preterm populations, in which mild IVH is more frequently observed, while severe IVH is associated with higher mortality and adverse neurodevelopmental outcomes [8], 9]. We also found that severe IVH was significantly more common among extremely preterm infants, affecting 21.9 % of this group, compared with 6 % of very preterm infants, while no cases of severe IVH were observed among late preterm infants. In addition, the overall frequency of IVH was highest among extremely preterm infants and decreased progressively with increasing gestational age. These findings suggest that both the frequency and severity of IVH increase with decreasing gestational age. Similar observations have been reported by Rysavy et al. and Ancel et al., who demonstrated that both the incidence and severity of IVH are greatest among the most immature infants [4], 5]. The marked fragility of the germinal matrix vasculature, immature cerebral autoregulation, and increased susceptibility to hemodynamic instability likely contribute to this increased vulnerability.

In our study, no significant differences were observed between the IVH and non-IVH groups with respect to pH, pCO_2_, hemoglobin, hematocrit, or standard base excess values. This finding suggests that isolated early indicators of acidosis, hypercapnia, or anemia may have limited value in identifying infants at risk for IVH. In contrast, lactate levels were significantly higher in infants with IVH and were independently associated with IVH in the blood gas model; however, this association became borderline in the exploratory integrated model including gestational age and hematological parameters. Lactate is widely regarded as a marker of systemic hypoperfusion and has been associated with impaired tissue perfusion and cerebral circulatory instability in neonates [10], 11]. In the blood gas model, each 1 mmol/L increase in lactate level was associated with an approximately 12 % increase in the odds of IVH. Elevated lactate levels may reflect hemodynamic instability, tissue hypoxia, and metabolic stress, all of which have been implicated in fluctuations of cerebral blood flow in preterm infants. Pellicer et al. reported an association between elevated lactate levels and cerebral hypoperfusion, while Gjerris et al. identified early lactate elevation as a marker of hemodynamic instability [10], 11]. Taken together, these findings suggest that elevated lactate is associated with IVH and support the need for further prospective studies evaluating the temporal relationship between systemic perfusion markers and IVH in preterm infants.

The finding that carboxyhemoglobin (COHb) levels were higher in the group without IVH is contrary to expectations based on the existing literature and does not suggest a clinically meaningful protective effect. Given the minimal difference between groups, the wide distribution of COHb values, and the lack of consistent evidence supporting COHb as a biomarker for IVH, the clinical significance of this finding appears limited. In addition, the presence of negative values and outliers suggests that measurement variability and technical factors may have influenced the results. Therefore, the observed association between COHb and IVH should be interpreted with caution. For this reason, COHb was not considered a clinically meaningful marker in the interpretation of the present findings.

In our study, significant differences in hematological parameters were observed between infants with and without IVH, including hemoglobin (Hgb), platelet count (PLT), mean platelet volume (MPV), eosinophil count, and the neutrophil-to-lymphocyte (N/L) ratio. Lower hemoglobin, platelet, and MPV values, together with higher N/L ratios in the IVH group, suggest that hematological and inflammatory balance may be associated with IVH development.

Lower hemoglobin levels and a progressive decline in hemoglobin values with increasing IVH severity may reflect impaired oxygen delivery and greater physiological vulnerability in preterm infants. Given the immaturity of cerebral autoregulatory mechanisms, reduced oxygen-carrying capacity may be associated with increased susceptibility to cerebral perfusion instability [12]. However, hemoglobin level was not independently associated with IVH in the multivariable analysis, suggesting that anemia may represent a marker of prematurity, clinical severity, and accompanying comorbidities rather than a direct contributor to IVH development [13].

Lower platelet counts were observed in infants with IVH and remained independently associated with IVH in multivariable analysis. For clinical interpretability, the platelet count association in the integrated model corresponds to an approximate OR of 0.82 for each 50 × 10^3^/µL increase in platelet count, indicating that higher platelet counts were associated with lower odds of IVH. This finding is consistent with the established role of platelets in maintaining hemostasis and vascular integrity. Thrombocytopenia has previously been associated with an increased risk of IVH in preterm infants, potentially reflecting impaired hemostatic capacity in the fragile germinal matrix vasculature [14].

One of the notable findings of our study was that MPV values were significantly lower in infants who developed IVH, and MPV decreased progressively with increasing IVH severity. MPV is considered an indirect marker of platelet activation and functional capacity. The independent association between lower MPV values and IVH suggests that platelet characteristics, in addition to platelet count, may be relevant in the development of IVH. Previous studies have similarly reported associations between platelet indices, neonatal bleeding tendency, and hemostatic function [15].

The neutrophil-to-lymphocyte (N/L) ratio was higher in infants with IVH and remained independently associated with IVH in multivariable analysis. The N/L ratio has been widely used as an accessible marker of systemic inflammation in neonatal studies [16]. Elevated N/L ratios may reflect intrauterine or postnatal inflammatory processes, which have been implicated in the development of IVH through mechanisms such as cytokine-mediated endothelial injury, increased vascular permeability, and disruption of germinal matrix integrity. Therefore, the observed association between elevated N/L ratios and IVH in our study is consistent with a potential contribution of inflammatory processes to IVH development.

Although eosinophil counts were lower in infants with IVH, they were not independently associated with IVH in the multivariable analysis. Eosinophils are known to play a role in immune regulation and modulation of inflammatory responses during the neonatal period; however, the clinical significance of this finding remains uncertain. The observed decrease in eosinophil counts may reflect immune suppression or a nonspecific stress response rather than a direct relationship with IVH development [17].

Overall, these findings suggest that IVH is associated not only with hemodynamic instability but also with alterations in hematological and inflammatory parameters. The independent associations observed for lower platelet count, lower MPV, and higher N/L ratio indicate that readily available laboratory parameters obtained early in the postnatal period may reflect clinical and biological processes related to IVH. Further prospective multicenter studies with standardized imaging and sampling protocols are needed to determine whether these parameters have true predictive value. Moreover, because no prespecified laboratory cut-off values were evaluated and no external validation was performed, these findings should not be used for bedside risk stratification or clinical decision-making at this stage.

A major strength of this study is the inclusion of a relatively large cohort of preterm infants from a tertiary neonatal intensive care unit. The evaluation of 356 preterm infants provided a relatively large sample for the assessment of multiple clinical and laboratory variables associated with IVH. In addition, the simultaneous analysis of initial blood gas and hematological parameters enabled a comprehensive evaluation of potential laboratory markers related to IVH. Finally, the identification of platelet count, MPV, and N/L ratio as independently associated variables in the integrated model, together with the borderline association of lactate, contributes to the growing body of evidence regarding laboratory parameters related to IVH in preterm infants.

This study has several limitations that should be considered when interpreting the findings. First, because of its retrospective design, data collection relied on existing medical records, which may have introduced variability in the completeness and timing of recorded measurements. In particular, the timing of admission blood gas and complete blood count sampling was not standardized and varied according to clinical workflow; therefore, exact postnatal sampling times could not be analyzed. Although several antenatal, perinatal, and postnatal variables were evaluated, residual confounding from unmeasured or incompletely recorded clinical factors cannot be excluded because of the retrospective design. In addition, MPV and some blood gas parameters may be influenced by preanalytical factors, including sampling source, anticoagulant exposure, sample handling, and time from sampling to analysis. These factors could not be fully controlled or verified for all patients because of the retrospective design.

Although laboratory parameters were obtained at admission, the first cranial ultrasonography was performed at a mean postnatal age of 3.73 days. Therefore, the temporal relationship between admission laboratory findings and the exact onset of IVH could not be definitively established. For this reason, the present findings should be interpreted as associations rather than evidence of true predictive ability.

The number of severe IVH cases was small, with only 14 infants having grade III–IV IVH. Therefore, the study was not sufficiently powered to perform robust multivariable analyses for severe IVH, and findings regarding IVH severity should be interpreted descriptively.

IVH cases were identified through retrospective medical record review and diagnostic coding. Although diagnostic records were reviewed, the possibility of misclassification cannot be completely excluded.

The single-center nature of the study may limit the generalizability of the findings. Differences in care protocols, resuscitation practices, ventilation strategies, and hemodynamic management across institutions may influence the frequency and severity of IVH.

Multiple pregnancies were analyzed at the infant level. However, pairwise concordance of IVH between co-twins could not be reliably assessed due to the retrospective structure of the dataset.

Finally, long-term neurodevelopmental outcomes were not evaluated, and the analysis was limited to data obtained during the neonatal period. Therefore, no conclusions can be drawn regarding the long-term neurodevelopmental consequences of IVH or the relationship between early laboratory parameters and subsequent neurodevelopmental impairment. Future prospective multicenter studies with standardized sampling protocols, standardized cranial ultrasonography timing, and long-term neurodevelopmental follow-up are needed to validate our findings and further clarify their clinical significance.

## Conclusions

In preterm infants, lower platelet counts, lower MPV values, and higher neutrophil-to-lymphocyte ratios were independently associated with IVH in the exploratory integrated model, while admission lactate showed a borderline association after adjustment for gestational age and hematological parameters. These findings suggest that hemodynamic, hematological, and inflammatory alterations may be related to IVH in this population. However, because of the retrospective design and the nonstandardized timing of cranial ultrasonography, these results should be interpreted as hypothesis-generating associations rather than validated predictive biomarkers. Prospective multicenter studies with standardized sampling protocols, early and serial cranial ultrasonography, external validation, and long-term neurodevelopmental follow-up are needed to determine their true predictive value and clinical relevance.
